# Impact of the COVID-19 pandemic on invasive pneumococcal disease in American Indian communities in the Southwest US

**DOI:** 10.1099/jmm.0.001983

**Published:** 2025-03-07

**Authors:** Catherine G. Sutcliffe, Shea Littlepage, Del Yazzie, George Brasinikas, Loretta Christensen, Shawnell Damon, Estar Denny, Sheri L. Dixon, Lindsay R. Grant, Marcella Harker-Jones, James McAuley, Pierrette Montanez, Dennie Parker, Alisa Reasonover, Amy Rice, Kristen Roessler, Eugene Romancito, Charis Salabye, Victoria M. Sergent, Brenna Simons-Petrusa, Valerie Tenequer, Polly Thompson, Minnie Tsingine, Robert C. Weatherholtz, Laura L. Hammitt

**Affiliations:** 1 Center for Indigenous Health, Johns Hopkins Bloomberg School of Public Health, Baltimore, Maryland, USA; 2 Navajo Epidemiology Center, Window Rock, Arizona, USA; 3 Gallup Indian Medical Center, Indian Health Service, Gallup, New Mexico, USA; 4 Indian Health Service, Rockville, MD, USA; 5 Navajo Area Indian Health Service, Window Rock, Arizona, USA; 6 Tséhootsooí Medical Center, Fort Defiance, Arizona, USA; 7 Arctic Investigations Program, Centers for Disease Control and Prevention, Anchorage, Alaska, USA; 8 Whiteriver Indian Hospital, Indian Health Service, Whiteriver, Arizona, USA; 9 Northern Navajo Medical Center, Indian Health Service, Shiprock, New Mexico, USA; 10 Crownpoint Health Care Facility, Indian Health Service, Crownpoint, New Mexico, USA; 11 Tuba City Regional Health Care Corporation, Tuba City, Arizona, USA; 12 Kayenta Health Center, Kayenta, Arizona, USA; 13 Chinle Comprehensive Health Care Facility, Indian Health Service, Chinle, Arizona, USA; 14 Winslow Indian Health Care Center, Winslow, Arizona, USA

**Keywords:** COVID-19, Indigenous health, invasive pneumococcal disease, surveillance

## Abstract

American Indian (AI) communities in the Southwest have a high burden of invasive pneumococcal disease (IPD) and COVID-19. Through laboratory-based surveillance, the impact of the pandemic on IPD incidence and serotype distribution was evaluated in two AI communities. IPD rates were lower during the pandemic (21.8 vs. 39.0/100 000 pre-pandemic). Rates increased in 2021 compared to 2020 but not to pre-pandemic levels. Cases with SARS-CoV-2 co-infection had a higher case fatality rate (45.2% vs. 17.6% without co-infection). No significant change in serotype distribution was observed. Continued surveillance in these communities is critical to understand the changing IPD burden as the pandemic evolves.

## Background

The emergence of the COVID-19 pandemic in 2020 and subsequent implementation of public health measures had a dramatic impact on the transmission and circulation of other pathogens, including respiratory viruses and bacteria. *Streptococcus pneumoniae* is an important cause of severe bacterial disease, including pneumonia, meningitis and sepsis, and often follows a viral respiratory infection. Despite initial concerns about increases in invasive pneumococcal disease (IPD) during the pandemic, rates of IPD largely declined globally in 2020 [1]. With the easing of pandemic restrictions, rates of IPD have increased in some settings [2].

American Indian and Alaska Native (AI/AN) individuals are disproportionately affected by both IPD and COVID-19. Despite high coverage of pneumococcal conjugate vaccines (PCVs) in AI/AN communities, IPD rates remained two to four times higher than the general U.S. population before the pandemic [3]. AI/AN communities were hit extremely hard by COVID-19, and nationally, AI/AN individuals are at increased risk of infection, hospitalization and death compared with white, non-Hispanic individuals [4]. In response, public health measures of varying degrees, including curfews, stay-at-home orders, school closures and mask mandates, were put in place with the goal of decreasing transmission of SARS-CoV-2.

Since the 1990s, the Johns Hopkins Center for Indigenous Health (JHCIH) has conducted surveillance to monitor IPD in two AI communities in the Southwest U.S. [5]. The goal of this analysis was to evaluate the impact of the first 2 years of the COVID-19 pandemic on the incidence and serotype distribution of IPD.

## Methods

### Study setting and population

The study was conducted in the Navajo Nation (~243 000 tribal members) and White Mountain Apache (WMA) Tribal lands (~17 000 tribal members). PCVs, including 7 (PCV7 : 4, 6B, 9V, 14, 18C, 19F, 23F) and 13 (PCV13: PCV7 and 1, 3, 5, 6A, 7F, 19A) serotypes, were introduced for routine use into the paediatric immunization schedule in 2000 and 2010, respectively, with rapid uptake; an estimated 85% of Navajo children aged 24–27 months received four doses of PCV13 in 2019 (https://www.ihs.gov/epi/immunization-and-vaccine-preventable-diseases/statistics-and-reports/). Coverage during the COVID-19 pandemic decreased, with only 74% of Navajo children aged 24–27 months receiving four doses of PCV13 in 2022.

Navajo Nation and the WMA Tribe detected their first COVID-19 cases on 17 March and 1 April 2020, respectively. Public health measures (e.g. stay-at-home orders, school closures, limits on gatherings and mask mandates) were implemented as a result. Both communities were more cautious about easing restrictions than surrounding areas, with mask mandates reinstated in the WMA Tribal lands during surges and most recently lifted in September 2022, and continuously in effect in Navajo Nation until January 2023 [6,7].

### Overview of surveillance and procedures

The JHCIH Active Bacterial Surveillance (ABS) programme was initiated in the mid-1990s to investigate invasive infections due to *Streptococcus pneumoniae*, *Haemophilus influenzae* and *Neisseria meningitidis*, and has been previously described [5]. Prospective, active, population-based and laboratory-based surveillance is conducted at the 24 Indian Health Service (IHS), Tribal and private facilities providing healthcare services to Tribal members.

Cases are identified through regular contact with laboratories. In addition, cases reported to the Navajo Epidemiology Center by the state health departments in New Mexico and Arizona, but not detected by JHCIH, are included. IPD cases are defined as *S. pneumoniae* cultured from a normally sterile body fluid collected from an AI individual living in or near the Navajo Nation or WMA Tribal lands. Information on case demographics, clinical presentation and treatment, and medical history is collected by medical record review.

Isolates are collected and sent to the Centers for Disease Control and Prevention (CDC) Arctic Investigations Programme in Alaska for serotyping. Serotyping is performed by slide agglutination and confirmed with the Quellung reaction using commercial group- and type-specific antisera (SSI Diagnostica, Staten Serum Institut, Copenhagen, Denmark). Isolates undergo antimicrobial susceptibility testing using a Microbroth dilution test (custom panel from Trek Diagnostic System, USA) and MICs are reported.

### Statistical analysis

For this analysis, the first 2 years of the pandemic (1 April 2020 to 31 March 2022) were compared with the 2 years preceding the pandemic (1 April 2018 to 31 March 2020). IPD incidence rates were calculated using annual case counts as numerators and the IHS User Population (all AI/AN people receiving IHS-funded healthcare services at least once at the facility during the preceding 3 years) as denominators. Annual incidence rates were calculated by pre-pandemic/pandemic year starting on 1 April of a given year, for all serotypes combined and for PCV13-type serotypes, and separately by age group (<5, 5–17, 18–49, 50–64 and ≥65 years). For analyses of vaccine-type incidence rates, missing serotype data were imputed by age group, assuming that isolates not serotyped had the same serotype distribution as those typed for a given era [2020–2022 : 27/111 (24.3%) missing; 2018–2020 : 14/202 (6.9%) missing]. Missing serotypes for analyses of individual serotypes were not imputed due to small numbers.

To evaluate the impact of the COVID-19 pandemic, incidence rates were also calculated for each era for all serotypes combined, PCV13-type serotypes and individual serotypes, both overall and separately by age group (<5, 18–49, 50–64 and ≥65 years). Incidence rate ratios between eras were compared using Poisson regression.

The serotype distribution and proportion of cases that were PCV13-type, PCV15/non-PCV13-type (22F, 33F), PCV20/non-PCV15-type (8, 10A, 11A, 12F, 15B/C) and non-vaccine-type were summarized overall and by age group for each era. For adults, the proportion of cases caused by serotypes included in the 21-valent PCV under development (PCV21 : 3, 6A, 7F, 8, 9N, 10A, 11A, 12F, 15A, 15B/C, 16F, 17F, 19A, 20, 22F, 23A, 23B, 24F, 31, 33F, 35B) and pneumococcal polysaccharide vaccine-type (PPSV23 : 1, 2, 3, 4, 5, 6B, 7F, 8, 9N, 9V, 10A, 11A, 12F, 14, 15B/C, 17F, 18C, 19A, 19F, 20, 22F, 23F, 33F) were also summarized. For PCV20, PCV21 and PPSV23, cross-protection between 15B and 15C was assumed.

The characteristics of cases between eras were compared using a chi-square test for categorical variables and the Wilcoxon rank-sum test for continuous variables. During the pandemic era, SARS-CoV-2 co-infection was defined as a positive test within 30 days before or after the IPD date. Results of antimicrobial resistance testing were summarized by era as the proportion resistant, intermediate and susceptible using published guidelines [8].

## Results

### Incidence rates and comparison between eras

**Fig. 1.**
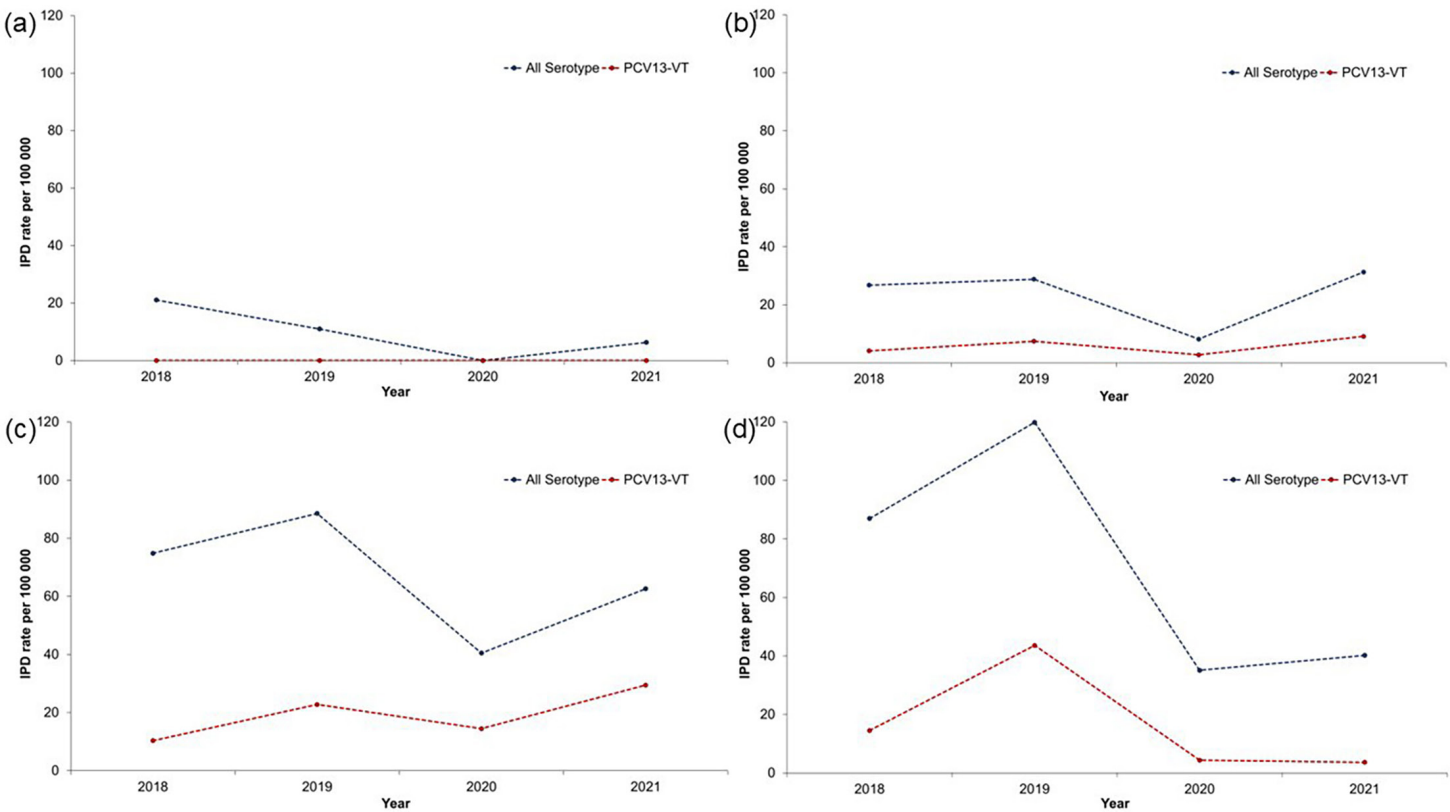
Incidence rate of all-type and PCV13-type IPD from April 2018 to March 2022 among (a) children <5 years, (**b**) adults 18–49 years, (**c**) adults 50–64 years and (d) adults ≥65 years. IPD, invasive pneumococcal disease; VT, vaccine-type. Note: The years start in April of the year indicated and end in March of the following year (e.g. ‘2018’ is April 2018 to March 2019 and ‘2019’ is April 2019 to March 2020).

When comparing eras, rates of all-serotype IPD were significantly lower during the pandemic (21.8 vs. 39.0/100 000; *P*<0.01; Fig. 1, Table 1). Rates were lower across all age groups (Table 1), although decreases were only statistically significant for adults 50–64 (51.5 vs. 81.7/100 000; *P*=0.02) and ≥65 (37.7 vs. 103.8/100 000; *P*<0.01) years. In the second year of the pandemic, the incidence of all-serotype IPD increased across age groups but remained lower than pre-pandemic levels (Fig. 1). Similar trends were found for rates of PCV13-type IPD overall and by age group (Fig. 1), although no cases were observed among children <5 years of age and few cases were observed among children 5–17 years of age in both eras.

**Table 1.** Incidence rate of all-type and PCV13-type IPD by era and age group, Navajo Nation and White Mountain Apache Tribal lands, April 2018 to March 2022

Age group (year)	Pre-pandemic		Pandemic		IRR (95% CI)	***P*-value**
	April 2018 to March 2020		April 2020 to March 2022			
	*N*	Rate*	*N*	Rate*		
**All-type**						
All ages	202	39.0 (33.8, 44.8)	111	21.8 (18.0, 26.3)	0.6 (0.4, 0.7)	<0.01
<5	6	16.1 (5.9, 35.1)	1	3.0 (0.1, 16.9)	0.2 (0, 1.6)	0.10
5–17	5	4.3 (1.4, 10.1)	0	0	0	0.04
18–49	62	27.8 (21.3, 35.6)	43	19.7 (14.2, 26.5)	0.7 (0.5, 1.1)	0.08
50–64	68	81.7 (63.5, 103.6)	43	51.5 (37.3, 69.4)	0.6 (0.4, 0.9)	0.02
≥65	61	103.8 (79.4, 133.3)	24	37.7 (24.2, 56.1)	0.4 (0.2, 0.6)	<0.01
**PCV13-type†**						
All ages	45	8.7 (6.3, 11.6)	34	6.7 (4.6, 9.3)	1.0 (0.6, 1.6)	0.89
<5	0	0	0	0	–	–
5–17	1	0.9 (0, 4.8)	0	0	0	1.00
18–49	13	5.8 (3.1, 10.0)	13	5.9 (3.2, 10.2)	1.0 (0.4, 2.4)	0.96
50–64	14	16.8 (9.2, 28.2)	18	21.6 (12.8, 34.1)	1.3 (0.6, 2.8)	0.49
≥65	17	28.9 (16.9, 46.3)	3	4.7 (1.0, 13.8)	0.2 (0, 0.6)	<0.01

* Rate per 100 000.

† PCV13-type includes serotypes 1, 3, 4, 5, 6A, 6B, 7F, 9V, 14, 18C, 19A, 19F and 23F.

CI, confidence interval; IRR, incidence rate ratio.

Rates of serotype-specific IPD decreased during the pandemic for most serotypes among children and adults (Tables S1 and S2, available in the online Supplementary Material). Among adults, significant decreases were found for serotypes 3, 22F, 9N and 16F. Among adults, increases in serotype-specific rates were found for serotypes 4, 19A, 6C and 34, although few cases of each serotype occurred and none of the increases were statistically significant.

### Serotype distribution

During the pandemic era, the proportion of cases that were PCV13-type, PCV15/non-PCV13-type, PCV20/non-PCV15-type and non-vaccine serotypes was 29.8%, 2.4%, 14.3% and 53.6%, respectively (Table S3; see Tables S4–S7 for results by age group). No significant change in the serotype distribution was found compared to the pre-pandemic era (22.3%, 9.6%, 18.1%, 50.0%, respectively; *P*=0.10).

### Characteristics of IPD cases and isolates

During the pre-pandemic and pandemic eras, 202 and 111 IPD cases were identified, respectively (Fig. S1, Tables S8–S10). In both eras, most cases were adults ≥50 years (pre-pandemic: 63.9%; pandemic: 60.4%) and male (61.4%; 68.5%), and most adult cases had an underlying condition (86.9%; 88.2%). The primary disease syndrome associated with IPD was pneumonia (79.2%; 74.8%).

During the pandemic era, most cases (90.1%) were hospitalized (Table S10), and among 100 individuals with available outcome data, 27 died from their illness (Table S11). Co-infection with SARS-CoV-2 was identified for 30.9% [*n*=34/110; median: 0 days between IPD and SARS-CoV-2 testing (range: −1 to 21])] of IPD cases, with a higher case fatality rate observed (45.2% vs. 17.6%; *P*=0.01). The proportion of cases hospitalized (83.2%; *P*=0.1) and dying from their illness (15.3%; *P*=0.02) was lower in the pre-pandemic era.

Most isolates were collected from blood (95.0%; 94.6%) (Table S12). Low levels of antibiotic resistance were identified (Table S13).

## Discussion

In these two AI communities in the Southwest, the onset of the COVID-19 pandemic was associated with a decline in IPD incidence across age groups to the lowest levels recorded since the surveillance programme began >30 years ago. As described in other settings around the world [1], this occurred alongside declines in disease caused by other respiratory pathogens, including influenza virus and respiratory syncytial virus [9]. Taken together, these data highlight that public health measures implemented in the first year of the pandemic (e.g. mask mandates and physical distancing) resulted in reductions in non-SARS-CoV-2 respiratory disease.

The resurgence of IPD has been described in many regions as pandemic restrictions eased, and some settings have reached or exceeded pre-pandemic levels [2,10]. Some AI/AN communities were more cautious in easing restrictions; for example, the Navajo Nation mask mandate remained in place through January 2023 [6]. In this study, rates of IPD were higher in 2021 than in 2020 but did not reach pre-pandemic levels, suggesting that COVID-19 public health measures continued to have an impact on *S. pneumoniae* disease.

Among children <5 years of age, no cases of PCV13-type IPD were detected from 2018 to 2021. During the pandemic, PCV13 coverage decreased from 85% to 74% among Navajo children 24–27 months of age. Data on pneumococcal carriage are not available in these communities during the pandemic, but other settings observed minor changes in carriage during the first years of the pandemic [11,12]. Low levels of carriage of PCV13-type serotypes persisted prior to the pandemic [13], so continued surveillance will be important to monitor for PCV13-type IPD among children.

Both before and during the pandemic, the majority of PCV13-type IPD in adults was caused by serotype 3. Of note, serotype 4 IPD, which was rare in the PCV era, increased during the pandemic era [5]. Increases in serotype 4 IPD were also recently reported from other settings, including CDC Active Bacterial Core sites in California, Colorado and New Mexico [14]. *S. pneumoniae* has been known to cause disease outbreaks, although they tend to occur in settings with lower vaccination rates [15] or specific population groups (e.g. persons experiencing homelessness) [14]. Cases of serotype 4 IPD in this study were concentrated among unvaccinated male adults 18–39 years of age, but other risk factors (e.g. homelessness) were not observed (data not shown). Further work is needed to investigate the changing epidemiology of serotype 4 in this and other settings and to ensure pneumococcal vaccination for eligible individuals.

In summary, a decline in IPD incidence was observed in the first year of the COVID-19 pandemic in AI communities in the Southwest U.S. Rates increased across age groups as public health measures were eased in the second year of the pandemic. Continued surveillance of IPD in these communities disproportionately affected by both COVID-19 and IPD will be critical to understanding the changing burden of disease with the continued easing of public health measures, increased circulation of other respiratory viruses and potential increases in susceptibility with lower pneumococcal vaccine coverage.

## Supplementary material

10.1099/jmm.0.001983 Uncited Supplementary Material 1.

## Abbreviations

ABS: active bacterial surveillance
AI: American Indian
AN: alaska native
IHS: Indian Health Service
IPD: invasive pneumococcal disease
JHCIH: Johns Hopkins Center for Indigenous Health
PCV: pneumococcal conjugate vaccines
WMA: white mountain apache
