# Supplementary material for: Impact of the COVID-19 pandemic on invasive pneumococcal disease in American Indian communities in the Southwest US
Source: J Med Microbiol. 2025 Mar 7;74(3):001983. doi: 10.1099/jmm.0.001983 (PMC12282234; doi:10.1099/jmm.0.001983)
Supplement: Uncited Supplementary Material 1. [file jmm-74-01983-s001.pdf]

Supplemental Material

Impact of the COVID-19 pandemic on invasive pneumococcal disease in Indigenous communities in the Southwest US

Catherine G. Sutcliffe, Shea Littlepage, Del Yazzie, George Brasinikas, Loretta Christensen, Shawnell Damon, Estar Denny, Sheri L. Dixon, Lindsay R. Grant, Marcella Harker-Jones, James McAuley, Pierrette Montanez, Dennie Parker, Alisa Reasonover, Amy Rice, Kristen Roessler, Eugene Romancito, Charis Salabye, Victoria M. Sergent, Brenna Simons-Petrusa, Valerie Tenequer, Polly Thompson, Minnie Tsingine, Robert C. Weatherholtz, Laura L. Hammitt

Supplemental Tables

Supplemental Table 1. Serotype-specific rates of IPD for children <5 years of age, Navajo Nation and White Mountain Apache Tribal lands, April 2018 – March 2022

Supplemental Table 2. Serotype-specific rates of IPD for adults ≥18 years of age, Navajo Nation and White Mountain Apache Tribal lands, April 2018 – March 2022

Supplemental Table 3. Serotype distribution of IPD cases, Navajo Nation and White Mountain Apache Tribal lands, April 2018 – March 2022

Supplemental Table 4. Serotype distribution of IPD cases <5 years of age, Navajo Nation and White Mountain Apache Tribal lands, April 2018 – March 2022

Supplemental Table 5. Serotype distribution of IPD cases ≥18 years of age, Navajo Nation and White Mountain Apache Tribal lands, April 2018 – March 2022

Supplemental Table 6. Serotype distribution of IPD cases 50-64 years of age, Navajo Nation and White Mountain Apache Tribal lands, April 2018 – March 2022

Supplemental Table 7. Serotype distribution of IPD cases ≥65 years of age, Navajo Nation and White Mountain Apache Tribal lands, April 2018 – March 2022

Supplemental Table 8. Characteristics of IPD cases, Navajo Nation and White Mountain Apache Tribal lands, April 2018 – March 2022

Supplemental Table 9. Pneumococcal vaccine history of IPD cases, Navajo Nation and White Mountain Apache Tribal lands, April 2018 – March 2022

Supplemental Table 10. Disease syndromes and outcomes associated with IPD cases, Navajo Nation and White Mountain Apache Tribal lands, April 2018 – March 2022

Supplemental Table 11. Characteristics of fatal IPD cases, Navajo Nation and White Mountain Apache Tribal lands, April 2018 – March 2022

Supplemental Table 12. Source of pneumococcal isolates, Navajo Nation and White Mountain Apache Tribal lands, April 2018 – March 2022

Supplemental Table 13. Antimicrobial resistance among pneumococcal isolates, Navajo Nation and White Mountain Apache Tribal lands, April 2018 – March 2022

Supplemental Figures

Supplemental Figure 1. Cumulative number of IPD cases by week and year, January 2015 to March 2022

Supplemental Table 1. Serotype-specific rates of IPD for children <5 years of age, Navajo Nation and White Mountain Apache Tribal lands, April 2018 – March 2022

	Pre-pandemic 04/2018-03/2020		Pandemic 04/2020-03/2022		IRR (95% CI)	p-value[c]
	N	Rate[a]	N	Rate[a]		
**PCV15/non-PCV13-type[b], n (%)**						
22F	1	2.7 (0.1, 15.0)	0	0	0 (0, 44.1)	0.53
33F	1	2.7 (0.1, 15.0)	0	0	0 (0, 44.1)	0.53
**Other, n (%)**						
7C	1	2.7 (0.1, 15.0)	0	0	0 (0, 44.1)	0.53
9N	1	2.7 (0.1, 15.0)	0	0	0 (0, 44.1)	0.53
15A	1	2.7 (0.1, 15.0)	0	0	0 (0, 44.1)	0.53
20	0	0	1	3.0 (0.1, 16.9)	-	-

CI: confidence interval; IPD: invasive pneumococcal disease; IRR: incidence rate ratio; PCV: pneumococcal conjugate vaccine

[a] rate per 100,000

[b] PCV15/non-PCV13-type includes serotypes 22F and 33F

[c] P-values comparing pandemic to pre-pandemic eras estimated using chi-square or Fisher's exact test

Supplemental Table 2. Serotype-specific rates of IPD for adults ≥18 years of age, Navajo Nation and White Mountain Apache Tribal lands, April 2018 – March 2022

	Pre-pandemic 04/2018-03/2020		Pandemic 04/2020-03/2022		IRR (95% CI)	p-value[e]
	N	Rate[a]	N	Rate[a]		
**PCV13-type[b], n (%)**						
3	30	8.2 (5.5, 11.7)	12	3.3 (1.7, 5.7)	0.4 (0.2, 0.8)	<0.01
4	4	1.1 (0.3, 2.8)	7	1.9 (0.8, 3.9)	1.7 (0.4, 8.1)	0.39
7F	1	0.3 (0, 1.5)	0	0 (0, 1.0)	0 (0, 38.9)	0.50
19A	2	0.5 (0.1, 2.0)	3	0.8 (0.2, 2.4)	1.5 (0.2, 17.9)	0.69
19F	4	1.1 (0.3, 2.8)	3	0.8 (0.2, 2.4)	0.7 (0.1, 4.4)	0.72
**PCV15/non-PCV13-type[c], n (%)**						
22F	14	3.8 (2.1, 6.4)	1	0.3 (0, 1.5)	0.1 (0, 0.5)	<0.01
33F	2	0.5 (0.1, 2.0)	1	0.3 (0, 1.5)	0.5 (0, 9.6)	0.62
**PCV20/non-PCV15-type[d], n (%)**						
8	15	4.1 (2.3, 6.8)	6	1.6 (0.6, 3.6)	0.4 (0.1, 1.1)	0.05
10A	5	1.4 (0.4, 3.2)	3	0.8 (0.2, 2.4)	0.6 (0.1, 3.1)	0.51
11A	6	1.6 (0.6, 3.6)	2	0.5 (0.1, 2.0)	0.3 (0, 1.9)	0.18
12F	2	0.5 (0.1, 2.0)	0	0 (0, 1.0)	0 (0, 5.3)	0.25
15B	2	0.5 (0.1, 2.0)	1	0.3 (0, 1.5)	0.5 (0, 9.6)	0.62
15C	1	0.3 (0, 1.5)	0	0 (0, 1.0)	0 (0, 38.9)	0.50
**Other serotypes, n (%)**						
6C	2	0.5 (0.1, 2.0)	3	0.8 (0.2, 2.4)	1.5 (0.2, 17.9)	0.69
7C	7	1.9 (0.8, 4.0)	3	0.8 (0.2, 2.4)	0.4 (0.1, 1.9)	0.23
9N	14	3.8 (2.1, 6.4)	2	0.5 (0.1, 2.0)	0.1 (0, 0.6)	<0.01
15A	4	1.1 (0.3, 2.8)	2	0.5 (0.1, 2.0)	0.5 (0, 3.5)	0.45
16F	8	2.2 (0.9, 4.3)	1	0.3 (0, 1.5)	0.1 (0, 0.9)	0.02
17F	3	0.8 (0.2, 2.4)	3	0.8 (0.2, 2.4)	1 (0.1, 7.4)	0.99
20	19	5.2 (3.1, 8.1)	13	3.6 (1.9, 6.1)	0.7 (0.3, 1.5)	0.29
21	1	0.3 (0, 1.5)	0	0 (0, 1.0)	0 (0, 38.9)	0.50
23A	8	2.2 (0.9, 4.3)	2	0.5 (0.1, 2.0)	0.2 (0, 1.2)	0.07
23B	1	0.3 (0, 1.5)	1	0.3 (0, 1.5)	1 (0, 78.3)	0.99
27	1	0.3 (0, 1.5)	0	0 (0, 1.0)	0 (0, 38.9)	0.50
31	7	1.9 (0.8, 4.0)	3	0.8 (0.2, 2.4)	0.4 (0.1, 1.9)	0.23
34	1	0.3 (0, 1.5)	2	0.5 (0.1, 2.0)	2 (0.1, 117.7)	0.63
35A	1	0.3 (0, 1.5)	0	0 (0, 1.0)	0 (0, 38.9)	0.50
35B	6	1.6 (0.6, 3.6)	6	1.6 (0.6, 3.6)	1 (0.3, 3.7)	0.99
35C	0	0 (0, 1.0)	1	0.3 (0, 1.5)	-	0.50
35F	1	0.3 (0, 1.5)	0	0 (0, 1.0)	0 (0, 38.9)	0.50
37	0	0 (0, 1.0)	1	0.3 (0, 1.5)	-	0.50
38	2	0.5 (0.1, 2.0)	1	0.3 (0, 1.5)	0.5 (0, 9.6)	0.62
NT	4	1.1 (0.3, 2.8)	0	0 (0, 1.0)	0 (0, 1.5)	0.06

CI: confidence interval; IPD: invasive pneumococcal disease; IRR: incidence rate ratio; PCV: pneumococcal conjugate vaccine

[a] rate per 100,000

[b] PCV13-type includes serotypes 1, 3, 4, 5, 6A, 6B, 7F, 9V, 14, 18C, 19A, 19F, 23F

[c] PCV15/non-PCV13-type includes serotypes 22F and 33F

[d] PCV20/non-PCV15-type includes serotypes 8, 10A, 11A, 12F, and 15B/C (cross-protection assumed between 15B and 15C)

[e] P-values comparing pandemic to pre-pandemic eras estimated using chi-square or Fisher's exact test

Supplemental Table 3. Serotype distribution of IPD cases, Navajo Nation and White Mountain Apache Tribal lands, April 2018 – March 2022

	Pre-pandemic 04/2018-03/2020	Pandemic 04/2020-03/2022	p-value[e]
Number of cases[a]	188 / 202	84 / 111	
Serotypes, n (%)			
**PCV13-type[b], n (%)**	**42 (22.3)**	**25 (29.8)**	0.10
3	30 (16.0)	12 (14.3)	
4	4 (2.1)	7 (8.3)	
7F	1 (0.5)	0 (0.0)	
19A	3 (1.6)	3 (3.6)	
19F	4 (2.1)	3 (3.6)	
**PCV15/non-PCV13-type[c], n (%)**	**18 (9.6)**	**2 (2.4)**	
22F	15 (8.0)	1 (1.2)	
33F	3 (1.6)	1 (1.2)	
**PCV20/non-PCV15-type[d], n (%)**	**34 (18.1)**	**12 (14.3)**	
8	17 (9.0)	6 (7.1)	
10A	5 (2.7)	3 (3.6)	
11A	6 (3.2)	2 (2.4)	
12F	2 (1.1)	0 (0.0)	
15B	2 (1.1)	1 (1.2)	
15C	2 (1.1)	0 (0.0)	
**Other serotypes, n (%)**	**94 (50.0)**	**45 (53.6)**	
6C	2 (1.1)	3 (3.6)	
7C	8 (4.3)	3 (3.6)	
9N	15 (8.0)	2 (2.4)	
15A	5 (2.7)	2 (2.4)	
16F	8 (4.3)	1 (1.2)	
17F	3 (1.6)	3 (3.6)	
20	19 (10.1)	14 (16.7)	
21	1 (0.5)	0 (0.0)	
23A	8 (4.3)	2 (2.4)	
23B	2 (1.1)	1 (1.2)	
27	1 (0.5)	0 (0.0)	
31	7 (3.7)	3 (3.6)	
34	1 (0.5)	2 (2.4)	
35A	1 (0.5)	0 (0.0)	
35B	6 (3.2)	6 (7.1)	
35C	0 (0.0)	1 (1.2)	
35F	1 (0.5)	0 (0.0)	
37	0 (0.0)	1 (1.2)	
38	2 (1.1)	1 (1.2)	
NT	4 (2.1)	0 (0.0)	

IPD: invasive pneumococcal disease; NT: non-typeable; PCV: pneumococcal conjugate vaccine

[a] Number of cases with available serotype data / Total number of cases

[b] PCV13-type includes serotypes 1, 3, 4, 5, 6A, 6B, 7F, 9V, 14, 18C, 19A, 19F, and 23F

[c] PCV15/non-PCV13-type includes serotypes 22F and 33F

[d] PCV20/non-PCV15-type includes serotypes 8, 10A, 11A, 12F, and 15B/C (cross-protection assumed between 15B and 15C)

[e] P-value comparing overall serotype distribution (bolded categories) between pandemic to pre-pandemic eras estimated using Fisher's exact test

Supplemental Table 4. Serotype distribution of IPD cases <5 years of age, Navajo Nation and White Mountain Apache Tribal lands, April 2018 – March 2022

	Pre-pandemic 04/2018-03/2020	Pandemic 04/2020-03/2022	p-value[e]
Number of cases[a]	5 / 6	1 / 1	
Serotypes, n (%)			
**PCV13-type[b], n (%)**	**0 (0.0)**	**0 (0.0)**	0.67
**PCV15/non-PCV13-type[c], n (%)**	**2 (40.0)**	**0 (0.0)**	
22F	1 (20.0)	0 (0.0)	
33F	1 (20.0)	0 (0.0)	
**PCV20/non-PCV15-type[d], n (%)**	**0 (0.0)**	**0 (0.0)**	
**Other serotypes, n (%)**	**3 (60.0)**	**1 (100)**	
7C	1 (20.0)	0 (0.0)	
9N	1 (20.0)	0 (0.0)	
15A	1 (20.0)	0 (0.0)	
20	0 (0.0)	1 (100)	

IPD: invasive pneumococcal disease; NT: non-typeable; PCV: pneumococcal conjugate vaccine

[a] Number of cases with available serotype data / Total number of cases

[b] PCV13-type includes serotypes 1, 3, 4, 5, 6A, 6B, 7F, 9V, 14, 18C, 19A, 19F, and 23F

[c] PCV15/non-PCV13-type includes serotypes 22F and 33F

[d] PCV20/non-PCV15-type includes serotypes 8, 10A, 11A, 12F, and 15B/C (cross-protection assumed between 15B and 15C)

[e] P-value comparing overall serotype distribution (bolded categories) pandemic to pre-pandemic eras estimated using Fisher's exact test

Supplemental Table 5. Serotype distribution of IPD cases ≥18 years of age, Navajo Nation and White Mountain Apache Tribal lands, April 2018 – March 2022

	Pre-pandemic 04/2018-03/2020	Pandemic 04/2020-03/2022	p-value[g]
Number of cases[a]	178 / 191	83 / 110	
Serotypes, n (%)			
**PCV13-type[b], n (%)**	**41 (23.0)**	**25 (30.1)**	0.16
3	30 (16.9)	12 (14.5)	
4	4 (2.2)	7 (8.4)	
7F	1 (0.6)	0 (0.0)	
19A	2 (1.1)	3 (3.6)	
19F	4 (2.2)	3 (3.6)	
**PCV15/non-PCV13-type[c], n (%)**	**16 (9.0)**	**2 (2.4)**	
22F	14 (7.9)	1 (1.2)	
33F	2 (1.1)	1 (1.2)	
**PCV20/non-PCV15-type[d], n (%)**	**31 (17.4)**	**12 (14.5)**	
8	15 (8.4)	6 (7.2)	
10A	5 (2.8)	3 (3.6)	
11A	6 (3.4)	2 (2.4)	
12F	2 (1.1)	0 (0.0)	
15B	2 (1.1)	1 (1.2)	
15C	1 (0.6)	0 (0.0)	
**Other serotypes, n (%)**	**90 (50.6)**	**44 (53.0)**	
6C	2 (1.1)	3 (3.6)	
7C	7 (3.9)	3 (3.6)	
9N	14 (7.9)	2 (2.4)	
15A	4 (2.2)	2 (2.4)	
16F	8 (4.5)	1 (1.2)	
17F	3 (1.7)	3 (3.6)	
20	19 (10.7)	13 (15.7)	
21	1 (0.6)	0 (0.0)	
23A	8 (4.5)	2 (2.4)	
23B	1 (0.6)	1 (1.2)	
27	1 (0.6)	0 (0.0)	
31	7 (3.9)	3 (3.6)	
34	1 (0.6)	2 (2.4)	
35A	1 (0.6)	0 (0.0)	
35B	6 (3.4)	6 (7.2)	
35C	0 (0.0)	1 (1.2)	
35F	1 (0.6)	0 (0.0)	
37	0 (0.0)	1 (1.2)	
38	2 (1.1)	1 (1.2)	
NT	4 (2.2)	0 (0.0)	
**PCV21-type[e], n (%)**	**150 (84.3)**	**62 (74.7)**	0.07
**PPSV23-type[f], n (%)**	**124 (69.7)**	**57 (68.7)**	0.87

IPD: invasive pneumococcal disease; NT: non-typeable; PCV: pneumococcal conjugate vaccine; PPSV: pneumococcal polysaccharide vaccine

[a] Number of cases with available serotype data / Total number of cases

[b] PCV13-type includes serotypes 1, 3, 4, 5, 6A, 6B, 7F, 9V, 14, 18C, 19A, 19F, and 23F

[c] PCV15/non-PCV13-type includes serotypes 22F and 33F

[d] PCV20/non-PCV15-type includes serotypes 8, 10A, 11A, 12F, and 15B/C (cross-protection assumed between 15B and 15C)

[e] PCV21-type includes serotypes 3, 6A, 7F, 8, 9N, 10A, 11A, 12F, 15A, 15B/C, 16F, 17F, 19A, 20, 22F, 23A, 23B, 24F, 31, 33F, and 35B (cross-protection assumed between 15B and 15C)

[f] PPSV23-type includes serotypes 1, 2, 3, 4, 5, 6B, 7F, 8, 9N, 9V, 10A, 11A, 12F, 14, 15B/C, 17F, 18C, 19A, 19F, 20, 22F, 23F, and 33F (cross-protection assumed between 15B and 15C)

[g] P-values comparing pandemic to pre-pandemic eras estimated using chi-square or Fisher's exact test

Supplemental Table 6. Serotype distribution of IPD cases 50-64 years of age, Navajo Nation and White Mountain Apache Tribal lands, April 2018 – March 2022

	Pre-pandemic 04/2018-03/2020	Pandemic 04/2020-03/2022	p-value[g]
Number of cases[a]	64 / 68	31 / 43	
Serotypes, n (%)			
**PCV13-type[b], n (%)**	**13 (20.3)**	**13 (41.9)**	0.05
3	11 (17.2)	8 (25.8)	
4	1 (1.6)	2 (6.5)	
7F	1 (1.6)	0 (0.0)	
19A	0 (0.0)	2 (6.5)	
19F	0 (0.0)	1 (3.2)	
**PCV15/non-PCV13-type[c], n (%)**	**5 (7.8)**	**0 (0.0)**	
22F	4 (6.3)	0 (0.0)	
33F	1 (1.6)	0 (0.0)	
**PCV20/non-PCV15-type[d], n (%)**	**11 (17.2)**	**2 (6.5)**	
8	3 (4.7)	2 (6.5)	
10A	4 (6.3)	0 (0.0)	
11A	3 (4.7)	0 (0.0)	
15B	1 (1.6)	0 (0.0)	
**Other serotypes, n (%)**	**35 (54.7)**	**16 (51.6)**	
6C	1 (1.6)	1 (3.2)	
7C	4 (6.3)	1 (3.2)	
9N	5 (7.8)	1 (3.2)	
15A	2 (3.1)	1 (3.2)	
16F	3 (4.7)	0 (0.0)	
17F	1 (1.6)	0 (0.0)	
20	4 (6.3)	6 (19.4)	
23A	2 (3.1)	0 (0.0)	
31	4 (6.3)	1 (3.2)	
34	1 (1.6)	1 (3.2)	
35B	3 (4.7)	2 (6.5)	
35C	0 (0.0)	1 (3.2)	
37	0 (0.0)	1 (3.2)	
38	2 (3.1)	0 (0.0)	
NT	3 (4.7)	0 (0.0)	
**PCV21-type[e], n (%)**	**52 (81.3)**	**23 (74.2)**	0.43
**PPSV23-type[f], n (%)**	**39 (60.9)**	**22 (71.0)**	0.34

IPD: invasive pneumococcal disease; NT: non-typeable; PCV: pneumococcal conjugate vaccine; PPSV: pneumococcal polysaccharide vaccine

[a] Number of cases with available serotype data / Total number of cases

[b] PCV13-type includes serotypes 1, 3, 4, 5, 6A, 6B, 7F, 9V, 14, 18C, 19A, 19F, and 23F

[c] PCV15/non-PCV13-type includes serotypes 22F and 33F

[d] PCV20/non-PCV15-type includes serotypes 8, 10A, 11A, 12F, and 15B/C (cross-protection assumed between 15B and 15C)

[e] PCV21-type includes serotypes 3, 6A, 7F, 8, 9N, 10A, 11A, 12F, 15A, 15B/C, 16F, 17F, 19A, 20, 22F, 23A, 23B, 24F, 31, 33F, and 35B (cross-protection assumed between 15B and 15C)

[f] PPSV23-type includes serotypes 1, 2, 3, 4, 5, 6B, 7F, 8, 9N, 9V, 10A, 11A, 12F, 14, 15B/C, 17F, 18C, 19A, 19F, 20, 22F, 23F, and 33F (cross-protection assumed between 15B and 15C)

[g] P-values comparing pandemic to pre-pandemic eras estimated using chi-square or Fisher's exact test

Supplemental Table 7. Serotype distribution of IPD cases ≥65 years of age, Navajo Nation and White Mountain Apache Tribal lands, April 2018 – March 2022

	Pre-pandemic 04/2018-03/2020	Pandemic 04/2020-03/2022	p-value[g]
Number of cases[a]	57 / 61	19 / 24	
Serotypes, n (%)			
**PCV13-type[b], n (%)**	**16 (28.1)**	**2 (10.5)**	0.29
3	14 (24.6)	2 (10.5)	
19A	1 (1.8)	0 (0.0)	
19F	1 (1.8)	0 (0.0)	
**PCV15/non-PCV13-type[c], n (%)**	**6 (10.5)**	**1 (5.3)**	
22F	6 (10.5)	0 (0.0)	
33F	0 (0.0)	1 (5.3)	
**PCV20/non-PCV15-type[d], n (%)**	**10 (17.5)**	**3 (15.8)**	
8	7 (12.3)	1 (5.3)	
10A	0 (0.0)	1 (5.3)	
11A	1 (1.8)	1 (5.3)	
12F	1 (1.8)	0 (0.0)	
15C	1 (1.8)	0 (0.0)	
**Other serotypes, n (%)**	**25 (43.9)**	**13 (68.4)**	
6C	0 (0.0)	2 (10.5)	
7C	3 (5.3)	2 (10.5)	
9N	2 (3.5)	0 (0.0)	
15A	1 (1.8)	1 (5.3)	
16F	2 (3.5)	1 (5.3)	
17F	0 (0.0)	2 (10.5)	
20	6 (10.5)	0 (0.0)	
23A	3 (5.3)	1 (5.3)	
23B	1 (1.8)	0 (0.0)	
31	2 (3.5)	1 (5.3)	
34	0 (0.0)	1 (5.3)	
35A	1 (1.8)	0 (0.0)	
35B	3 (5.3)	2 (10.5)	
NT	1 (1.8)	0 (0.0)	
**PCV21-type[e], n (%)**	**51 (89.5)**	**14 (73.7)**	0.13
**PPSV23-type[f], n (%)**	**40 (70.2)**	**8 (42.1)**	0.03

IPD: invasive pneumococcal disease; NT: non-typeable; PCV: pneumococcal conjugate vaccine; PPSV: pneumococcal polysaccharide vaccine

[a] Number of cases with available serotype data / Total number of cases

[b] PCV13-type includes serotypes 1, 3, 4, 5, 6A, 6B, 7F, 9V, 14, 18C, 19A, 19F, 23F

[c] PCV15/non-PCV13-type includes serotypes 22F and 33F

[d] PCV20/non-PCV15-type includes serotypes 8, 10A, 11A, 12F, and 15B/C (cross-protection assumed between 15B and 15C)

[e] PCV21-type includes serotypes 3, 6A, 7F, 8, 9N, 10A, 11A, 12F, 15A, 15B/C, 16F, 17F, 19A, 20, 22F, 23A, 23B, 24F, 31, 33F, and 35B (cross-protection assumed between 15B and 15C)

[f] PPSV23-type includes serotypes 1, 2, 3, 4, 5, 6B, 7F, 8, 9N, 9V, 10A, 11A, 12F, 14, 15B/C, 17F, 18C, 19A, 19F, 20, 22F, 23F, and 33F (cross-protection assumed between 15B and 15C)

[g] P-values comparing pandemic to pre-pandemic eras estimated using chi-square or Fisher's exact test

Supplemental Table 8. Characteristics of IPD cases, Navajo Nation and White Mountain Apache Tribal lands, April 2018 - March 2022

	Pre-pandemic 04/2018-03/2020	Pandemic 04/2020-03/2022	p-value[a]
Number of cases	202	111	
Surveillance site, n (%)			
Navajo Nation	178 (88.1)	95 (85.6)	0.60
White Mountain Apache	24 (11.9)	16 (14.4)	
Age (years), median (IQR)	57.4 (43.1 to 69.6)	52.8 (42.1 to 63.9)	0.26
Age group (years), n (%)			0.16
<2	4 (2.0)	1 (0.9)	
2-4	2 (1.0)	0 (0.0)	
5-7	5 (2.5)	0 (0.0)	
18-49	62 (30.7)	43 (38.7)	
50-64	68 (33.7)	43 (38.7)	
≥65	61 (30.2)	24 (21.6)	
Sex – female, n (%)	78 (38.6)	35 (31.5)	0.22
Underlying conditions, n (%)[b]			
Alcohol abuse	77 (41.6)	57 (51.8)	0.09
Diabetes mellitus	78 (42.2)	47 (43.1)	0.90
Cirrhosis	35 (18.9)	23 (21.1)	0.65
Congestive heart failure	24 (13.0)	10 (9.2)	0.35
Asthma	26 (14.1)	7 (6.4)	0.06
Chronic renal failure	24 (13.0)	9 (8.2)	0.25
Malignancy	19 (10.3)	10 (9.1)	0.84
Immunosuppression	17 (9.2)	8 (7.3)	0.67
Smoking	13 (7.0)	11 (10.0)	0.39
Immunodeficiency	8 (4.3)	4 (3.7)	1.00
COPD	8 (4.3)	3 (2.7)	0.75
HIV/AIDS	2 (1.1)	5 (4.5)	0.11
Transplant	0 (0.0)	0 (0.0)	-
None	25 (13.1)	13 (11.8)	0.86

IPD: invasive pneumococcal disease; IQR: interquartile range

[a] P-values comparing pandemic to pre-pandemic eras estimated using chi-square or Fisher's exact test for categorical variables and Wilcoxon rank-sum test for continuous variables.

[b] Underlying conditions assessed by medical chart review and reported for adults only (pre-pandemic era: n=186/191; pandemic era: n=110/110). Underlying conditions were restricted to those identified by the Advisory Committee on Immunization Practices (ACIP) as warranting administration of pneumococcal vaccines [1].

Supplemental Table 9. Pneumococcal vaccine history of IPD cases, Navajo Nation and White Mountain Apache Tribal lands, April 2018 – March 2022

	Pre-pandemic	Pandemic	p-value[a]
	04/2018-03/2020	04/2020-03/2022	
**Age group: <5 years**			
Number of cases[b]	5 / 6	1 / 1	
Number of PCV doses received, n (%)			0.67
0	1 (20.0)	0 (0.0)	
1	0 (0.0)	1 (100)	
2	1 (20.0)	0 (0.0)	
3	0 (0.0)	0 (0.0)	
4	2 (40.0)	0 (0.0)	
5	1 (20.0)	0 (0.0)	
Fully vaccinated for age[c], n (%)	3 (60.0)	0 (0.0)	0.50
**Age group: 5-17 years**			
Number of cases[b]	5 / 5	0	
Number of PCV doses received, n (%)			
3	1 (20.0)	-	-
4	2 (40.0)	-	-
5	2 (40.0)	-	-
**Age group: 18-49 years**			
Number of cases[b]	57 / 62	41 / 43	
Number of PCV doses received, n (%)			
0	54 (94.7)	38 (92.7)	0.92
1	1 (1.8)	1 (2.4)	
2	1 (1.8)	1 (2.4)	
3	0 (0.0)	1 (2.4)	
4	1 (1.8)	0 (0.0)	
Number of PPSV23 doses received, n (%)			
0	36 (63.2)	28 (68.3)	0.83
1	18 (31.6)	10 (24.4)	
2	2 (3.5)	2 (4.9)	
3	1 (1.8)	1 (2.4)	
**Age group: 50-64 years**			
Number of cases[b]	60 / 68	42 / 43	
Number of PCV doses received, n (%)			
0	53 (88.3)	38 (90.5)	0.61
1	0 (0.0)	1 (2.4)	
2	2 (3.3)	1 (2.4)	
3	4 (6.7)	1 (2.4)	
4	0 (0.0)	1 (2.4)	
5	1 (1.7)	0 (0.0)	
Number of PPSV23 doses received, n (%)			
0	20 (33.3)	15 (35.7)	0.86
1	27 (45.0)	18 (42.9)	
2	7 (11.7)	6 (14.3)	
3	5 (8.3)	2 (4.8)	
4	0 (0.0)	1 (2.4)	
5	1 (1.7)	0 (0.0)	
**Age group: ≥65 years**			
Number of cases[b]	56 / 61	24 / 24	
Number of PCV doses received, n (%)			
0	12 (21.4)	8 (33.3)	0.43
1	2 (3.6)	0 (0.0)	
2	11 (19.6)	8 (33.3)	
3	12 (21.4)	2 (8.3)	
4	11 (19.6)	3 (12.5)	
5	8 (14.3)	3 (12.5)	

Number of PPSV23 doses received, n (%)			
0	5 (8.9)	2 (8.3)	0.58
1	5 (8.9)	3 (12.5)	
2	13 (23.2)	10 (41.7)	
3	13 (23.2)	3 (12.5)	
4	12 (21.4)	3 (12.5)	
5	8 (14.3)	3 (12.5)	

IPD: invasive pneumococcal disease; PCV: pneumococcal conjugate vaccine; PPSV: pneumococcal polysaccharide vaccine

[a] P-values comparing pandemic to pre-pandemic eras estimated using chi-square or Fisher's exact test

[b] Number of cases with available data on vaccine history / total number of cases

[c] Vaccination status was defined based on the age of the cases according to ACIP schedules, including catch-up schedules [2].

Supplemental Table 10. Disease syndromes and outcomes associated with IPD cases, Navajo Nation and White Mountain Apache Tribal lands, April 2018 – March 2022

	Pre-pandemic 04/2018-03/2020	Pandemic 04/2020-03/2022	p-value[a]
**All ages**			
Number of cases	202	111	
Disease syndrome, n (%)[b]			
Pneumonia	160 (79.2)	83 (74.8)	0.40
Sepsis	98 (48.5)	60 (54.1)	0.41
Meningitis	12 (5.9)	2 (1.8)	0.15
Cellulitis	3 (1.5)	5 (4.5)	0.14
Bacteremia (alone)	5 (2.5)	3 (2.7)	1.00
Peritonitis	1 (0.5)	3 (2.7)	0.13
STSS	3 (1.5)	0 (0.0)	0.56
Osteomyelitis	0 (0.0)	1 (0.9)	0.36
Arthritis (septic)	0 (0.0)	1 (0.9)	0.36
Pericarditis	1 (0.5)	0 (0.0)	1.00
Hospitalized, n (%)	168 (83.2)	100 (90.1)	0.10
Died, n (%)[c]	29 (15.3)	27 (27.0)	0.02
**Age group: <5 years**			
Number of cases	6	1	
Disease syndrome, n (%)[b]			
Pneumonia	4 (66.7)	0 (0.0)	0.43
Sepsis	1 (16.7)	0 (0.0)	1.00
Meningitis	1 (16.7)	0 (0.0)	1.00
Cellulitis	0 (0.0)	0 (0.0)	-
Bacteremia (alone)	0 (0.0)	0 (0.0)	-
Peritonitis	0 (0.0)	0 (0.0)	-
STSS	0 (0.0)	0 (0.0)	-
Osteomyelitis	0 (0.0)	1 (100.0)	0.14
Arthritis (septic)	0 (0.0)	0 (0.0)	-
Pericarditis	0 (0.0)	0 (0.0)	-
Hospitalized, n (%)	4 (66.7)	1 (100.0)	1.00
Died, n (%)[c]	0 (0.0)	0 (0.0)	-
**Age group: ≥18 years**			
Number of cases	191	110	
Disease syndrome, n (%)[b]			
Pneumonia	153 (80.1)	83 (75.5)	0.38
Sepsis	94 (49.2)	94 (49.2)	0.40
Meningitis	10 (5.2)	10 (5.2)	0.22
Cellulitis	3 (1.6)	3 (1.6)	0.15
Bacteremia (alone)	5 (2.6)	5 (2.6)	1.00
Peritonitis	1 (0.5)	1 (0.5)	0.14
STSS	3 (1.6)	3 (1.6)	0.30
Osteomyelitis	0 (0.0)	0 (0.0)	-
Arthritis (septic)	0 (0.0)	0 (0.0)	0.37
Pericarditis	1 (0.5)	1 (0.5)	1.00
Hospitalized, n (%)	159 (83.2)	159 (83.2)	0.11
Died, n (%)[c]	29 (16.5)	22 (25.6)	0.08

IPD: invasive pneumococcal disease; STSS: Streptococcal toxic shock syndrome

[a] P-values comparing pandemic to pre-pandemic eras estimated using chi-square or Fisher's exact test

[b] Multiple categories may be selected

[c] Proportions are based on the number of cases with available data on vital outcomes (pre-pandemic era: n=189/202 all ages, n=6/6 children <5 years and 178/191 adults ≥18 years; pandemic era: n=100/111 all ages, n=1/1 children <5 years and 99/110 adults ≥18 years)

Supplemental Table 11. Characteristics of fatal IPD cases, Navajo Nation and White Mountain Apache Tribal lands, April 2018 – March 2022

Characteristic	Pre-pandemic 04/2018-03/2020	Pandemic 04/2020-03/2022
Number of fatal cases	30	27
Surveillance site, n (%)		
Navajo Nation	26 (86.7)	23 (85.2)
White Mountain Apache	4 (13.3)	4 (14.8)
Age (years), median (IQR)	62.8 (51.9 to 78.0)	50.9 (44.0 to 56.3)
Age group (years), n (%)		
0-17	0 (0.0)	0 (0.0)
18-49	7 (23.3)	11 (40.7)
50-64	9 (30.0)	13 (48.1)
≥65	14 (46.7)	3 (11.1)
Sex, n (%)		
Female	12 (40.0)	8 (29.6)
Male	18 (60.0)	19 (70.4)
Hospitalized, n (%)	26 (86.7)	24 (88.9)
Serotype, n (%)[a]		
3	8 (28.6)	2 (10.5)
7C	0 (0.0)	2 (10.5)
8	2 (7.1)	0 (0.0)
9N	4 (14.3)	2 (10.5)
10A	1 (3.6)	0 (0.0)
11A	0 (0.0)	1 (5.3)
15A	1 (3.6)	0 (0.0)
15C	1 (3.6)	0 (0.0)
17F	1 (3.6)	0 (0.0)
19A	0 (0.0)	1 (5.3)
19F	1 (3.6)	0 (0.0)
20	1 (3.6)	3 (15.8)
21	1 (3.6)	0 (0.0)
22F	4 (14.3)	0 (0.0)
23A	0 (0.0)	1 (5.3)
31	2 (7.1)	1 (5.3)
35B	1 (3.6)	4 (21.1)
35C	0 (0.0)	1 (5.3)
38	0 (0.0)	1 (5.3)
Disease syndrome, n (%)		
Pneumonia	25 (83.3)	23 (85.2)
Sepsis	20 (66.7)	16 (59.3)
Meningitis	0 (0.0)	0 (0.0)
Cellulitis	1 (3.3)	1 (3.7)
Bacteremia (alone)	0 (0.0)	1 (3.7)
Peritonitis	0 (0.0)	1 (3.7)
STSS	2 (6.7)	0 (0.0)
Concurrent COVID-19 (within 30 days), n (%)[b]	-	14 (51.9)

IPD: invasive pneumococcal disease; IQR: interquartile range; STSS: Streptococcal toxic shock syndrome

[a] Serotype data unavailable for some cases (pre-pandemic era: 2/30 and pandemic era: 8/27), percentages are out of those with available serotypes

[b] Unknown for 1 case

Supplemental Table 12. Source of pneumococcal isolates, Navajo Nation and White Mountain Apache Tribal lands, April 2018 – March 2022

	Pre-pandemic 04/2018-03/2020	Pandemic 04/2020-03/2022	p-value[a]
**All ages**			
Number of cases	202	111	
Body source, n (%)			
Blood	192 (95.0)	105 (94.6)	0.17
Blood/cerebrospinal fluid	6 (3.0)	1 (0.9)	
Blood/ear	0 (0.0)	1 (0.9)	
Blood/joint fluid	0 (0.0)	1 (0.9)	
Blood/Peritoneal fluid	0 (0.0)	1 (0.9)	
Blood/Pleural fluid	2 (1.0)	0 (0.0)	
Bone	0 (0.0)	1 (0.9)	
Cerebrospinal fluid	1 (0.5)	1 (0.9)	
Joint fluid	0 (0.0)	0 (0.0)	
Pleural fluid	1 (0.5)	0 (0.0)	
**Age group: <5 years**			
Number of cases[a]	6	1	
Body source, n (%)			
Blood	5 (83.3)	0 (0.0)	0.29
Blood/cerebrospinal fluid	0 (0.0)	0 (0.0)	
Blood/ear	0 (0.0)	0 (0.0)	
Blood/joint fluid	0 (0.0)	0 (0.0)	
Blood/Peritoneal fluid	0 (0.0)	0 (0.0)	
Blood/Pleural fluid	0 (0.0)	0 (0.0)	
Bone	0 (0.0)	1 (100.0)	
Cerebrospinal fluid	1 (16.7)	0 (0.0)	
Joint fluid	0 (0.0)	0 (0.0)	
Pleural fluid	0 (0.0)	0 (0.0)	
**Age group: ≥18 years**			
Number of cases	191	110	
Body source, n (%)			
Blood	182 (95.3)	105 (95.5)	0.12
Blood/cerebrospinal fluid	6 (3.1)	1 (0.9)	
Blood/ear	0 (0.0)	1 (0.9)	
Blood/joint fluid	0 (0.0)	1 (0.9)	
Blood/Peritoneal fluid	0 (0.0)	1 (0.9)	
Blood/Pleural fluid	2 (1.0)	0 (0.0)	
Bone	0 (0.0)	0 (0.0)	
Cerebrospinal fluid	0 (0.0)	1 (0.9)	
Joint fluid	0 (0.0)	0 (0.0)	
Pleural fluid	1 (0.5)	0 (0.0)	

[a] P-values comparing pandemic to pre-pandemic eras estimated using Fisher's exact test

Supplemental Table 13. Antimicrobial resistance among pneumococcal isolates, Navajo Nation and White Mountain Apache Tribal lands, April 2018 – March 2022

	Pre-pandemic 04/2018-03/2020	Pandemic 04/2020-03/2022	p-value[b]
Number of isolates[a]	155 / 202	74 / 111	
Penicillin (non-meningitis), n (%)			1.00
Susceptible	155 (100.0)	74 (100.0)	
Intermediate	0 (0.0)	0 (0.0)	
Resistant	0 (0.0)	0 (0.0)	
Penicillin (meningitis), n (%)			0.48
Susceptible	150 (96.8)	70 (94.6)	
Intermediate	0 (0.0)	0 (0.0)	
Resistant	5 (3.2)	4 (5.4)	
Erythromycin, n (%)			0.03
Susceptible	145 (93.5)	63 (85.1)	
Intermediate	0 (0.0)	2 (2.7)	
Resistant	10 (6.5)	9 (12.2)	
Trimethoprim/Sulfamethoxazole, n (%)			0.37
Susceptible	137 (88.4)	61 (82.4)	
Intermediate	11 (7.1)	9 (12.2)	
Resistant	7 (4.5)	4 (5.4)	
Vancomycin, n (%)			1.00
Susceptible	155 (100.0)	74 (100.0)	
Intermediate	0 (0.0)	0 (0.0)	
Resistant	0 (0.0)	0 (0.0)	
Levofloxacin, n (%)			1.00
Susceptible	155 (100.0)	74 (100.0)	
Intermediate	0 (0.0)	0 (0.0)	
Resistant	0 (0.0)	0 (0.0)	
Ceftriaxone (non-meningitis), n (%)			1.00
Susceptible	155 (100.0)	74 (100.0)	
Intermediate	0 (0.0)	0 (0.0)	
Resistant	0 (0.0)	0 (0.0)	
Ceftriaxone (meningitis), n (%)			0.40
Susceptible	113 (100.0)	73 (98.6)	
Intermediate	0 (0.0)	1 (1.4)	
Resistant	0 (0.0)	0 (0.0)	
Meropenem, n (%)			0.16
Susceptible	113 (100.0)	72 (97.3)	
Intermediate	0 (0.0)	2 (2.7)	
Resistant	0 (0.0)	0 (0.0)	
Chloramphenicol, n (%)			1.00
Susceptible	155 (100.0)	74 (100.0)	
Intermediate	0 (0.0)	0 (0.0)	
Resistant	0 (0.0)	0 (0.0)	

[a] Number of isolates with available antimicrobial resistance testing data / total number of cases.

[b] P-values comparing pandemic to pre-pandemic eras estimated using chi-square or Fisher's exact test

Supplemental Figure 1. Cumulative number of IPD cases by week and year, Navajo Nation and White Mountain Apache Tribal lands, April 2018 to March 2022

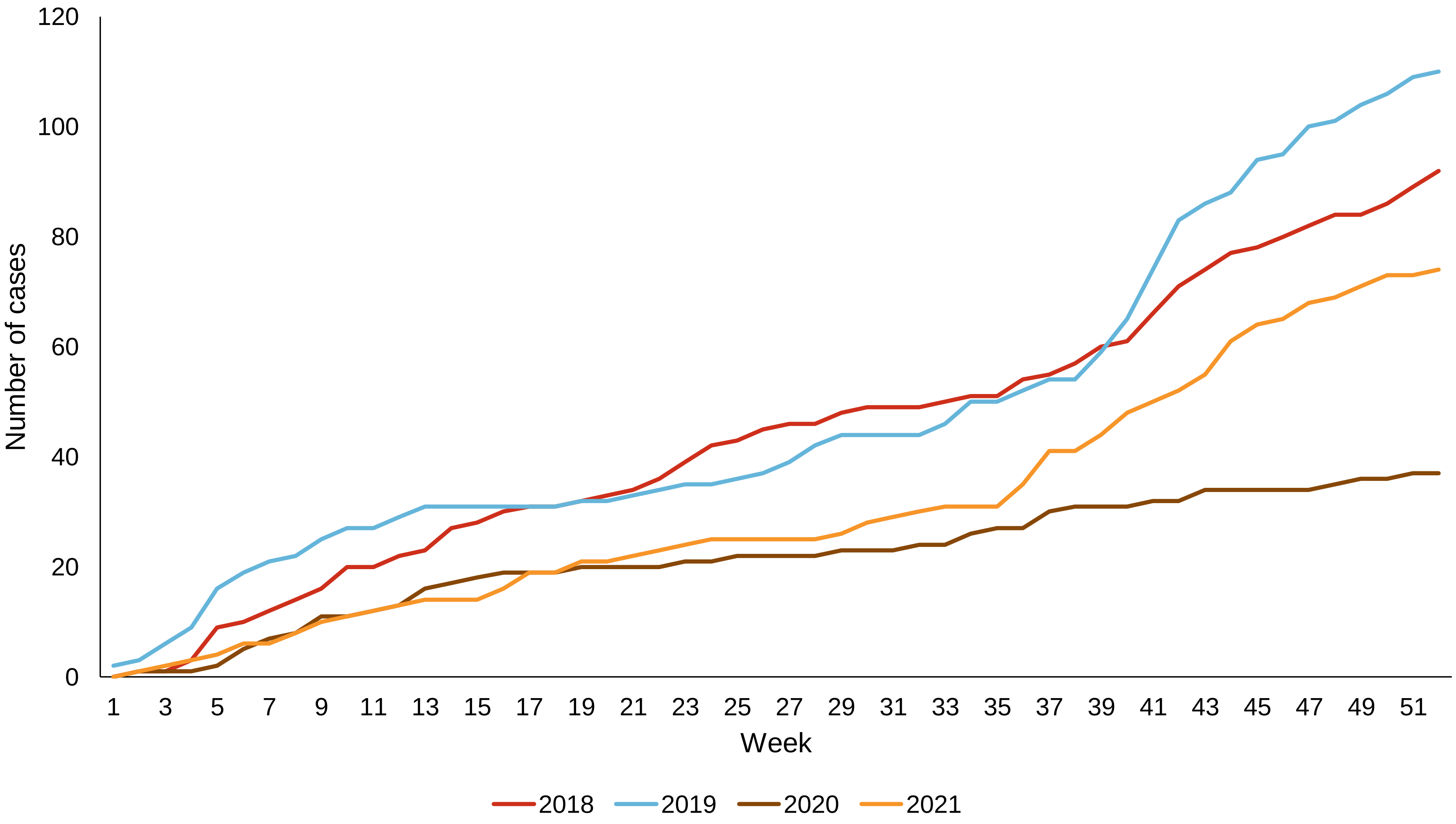

Note: The years start in April of the year indicated and end in March of the following year (e.g., '2018' is April 2018 to March 2019, '2019' is April 2019 to March 2020, etc.).

References

1. Kobayashi M, Farrar JL, Gierke R, et al. Use of 15-Valent Pneumococcal Conjugate Vaccine Among U.S. Children: Updated Recommendations of the Advisory Committee on Immunization Practices - United States, 2022. MMWR Morb Mortal Wkly Rep **2022**; 71 (37): 1174-81.

2. Centers for Disease Control and Prevention. Child and Adolescent Immunization Schedule. Recommendations for Ages 18 Years or Younger, United States, 2024. Available at: https://www.cdc.gov/vaccines/hcp/imz-schedules/child-adolescent-catch-up.html#table-2. Accessed October 4 2024.
